# Supplementary material for: Does decision confidence reflect effort?
Source: PLoS One. 2023 Feb 16;18(2):e0278617. doi: 10.1371/journal.pone.0278617 (PMC9934378; doi:10.1371/journal.pone.0278617)
Supplement: S1 File — (PDF) [file pone.0278617.s001.pdf]

Supplementary Material

Hagura, Esmaily & Bahrami.

Does decision confidence reflect effort?

Table S1. details of statistical results in behavioural data (Figure 1):

Response	Regressors	Estimate	SE	CI	tStat	p-value	Total Number
Accuracy	Coherency	.83	.06	[.71 .96]	13.3	<.001	9120
	Force	.0016	.004	[-.006 .009]	.41	.68	9120
Confidence	Coherency	1.73	.03	[1.67 1.8]	59.2	<.001	9120
	Force	.003	.002	[-.001 .006]	1.4	.16	9120
RT	Coherency	.8	.017	[.76 .83]	46.3	<.001	9120
	Force	.003	.001	[0.001 .005]	3.47	<.001	9120

Pilot experiment

Prior to the main experiment, a pilot experiment was performed to obtain the statistical hypothesis, and to calculate the sample size. 15 participants (5 females, mean age: 22.4, s.d.: 1.6 (20-26)) volunteered, but 1 was excluded from the analysis, due to the high proportion of non-responding trials.

The basic procedure was equivalent to the main experiment; participants judged the direction of a random-dot motion while holding a manipulandum. The only difference was that, instead of two force levels (0N and 6N), three force levels (0N, 2.5N, and 5N) were set. Participants performed 10 blocks, with each block consisting of 60 trials (20 trials per force level). This resulted in 200 trials per different force levels.

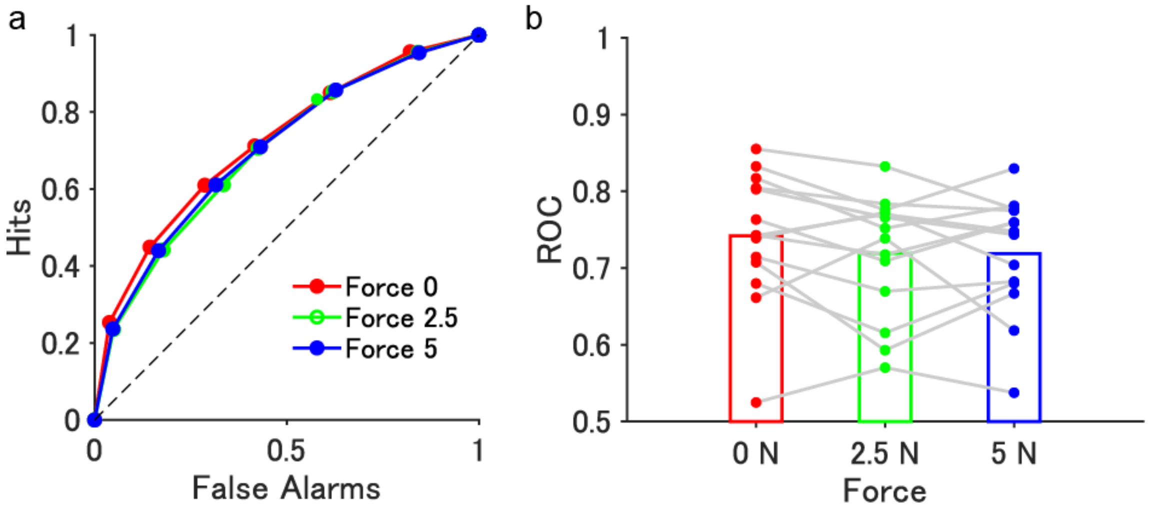

Figure S1. The results of the pilot experiment. **a**. ROC curves for the three conditions. **b**. Metacognitive accuracy for each condition. The accuracy became lower under the highest level of force compared to when no-force was applied.

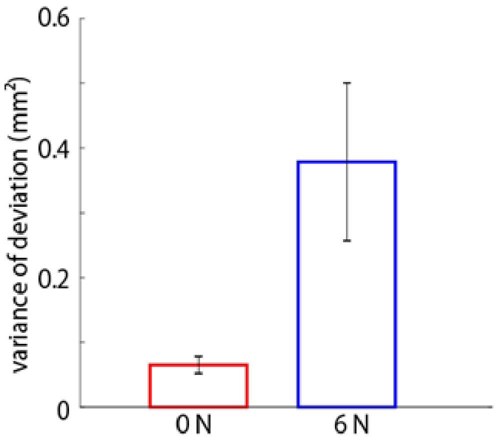

Figure S2. Variance of the handle position during the deliberation period of the decision (mm^2). Variance was calculated in the axis of the force direction. Variance was significantly higher for the force condition compared to the no-force condition ($t(19)=2.56$, $p=0.019$), indicating the higher signal (i.e. motor command) dependent noise in the force condition.

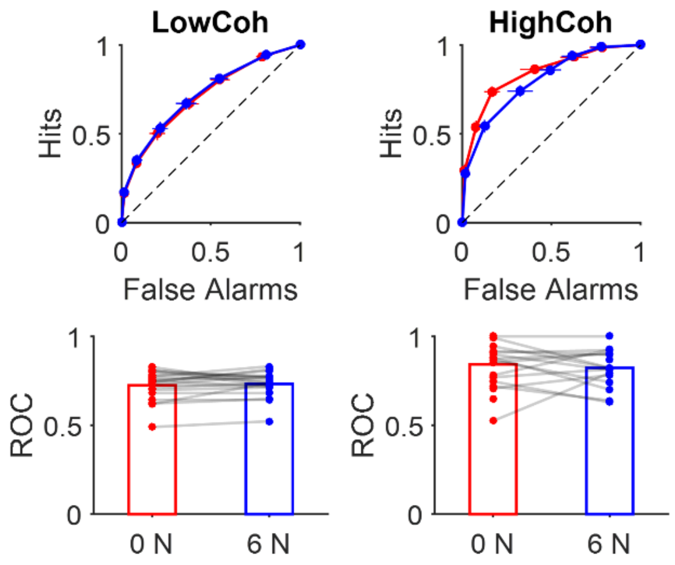

Figure S3. Metacognitive accuracy (ROC) calculated for each low (3.2, 6.4%) and high (12.8, 25.6 and 51.2%) motion coherence level. In both cases, the metacognitive accuracy did not significantly differ between the force and no-force condition (low; $t(19)=0.69$, $p=0.50$, high; $t(19)=0.56$, $p=0.59$).
