# Supplementary material for: Does decision confidence reflect effort?
Source: PLoS One. 2023 Feb 16;18(2):e0278617. doi: 10.1371/journal.pone.0278617 (PMC9934378; doi:10.1371/journal.pone.0278617)
Supplement: Attachment — Submitted filename: Revision2_Response_to_Reviewers.docx [file pone.0278617.s003.docx]

### **Response to the Reviewers**

### Reviewer #2

Comment1: *I appreciate the effort made by the authors to improve the manuscript. I think it has gain in clarity. Still, few remaining comments:*

*Reply 2: I understand that the pilot experiment was only performed as an exploratory step before performing the experiment of the main paper. While this is completely fine and I am happy that it is now clearly stated in the text, I am still not convinced of using the t-test to compare the data. Under the experimental conditions that the authors are using, it is not correct to use such test. Thus, I would suggest to include the data as it is (in Fig S1), but to not include the result of a t-test. The authors could anyway discuss the tendency that they observed. Indeed, it should be clearly stated in lines 173-175 that there is a tendency.*

**Reply1: Following reviewer’s suggestions, we have revised the Supplementary Materials. First, we omitted the description of t-test from the legend of Figure S1, and also, deleted the asterisk (indicator of significance) from Figure S1b.**

Comment2: *Throughout manuscript, p values are reported by not the specific statistical test used. For instance, lines number 291, 295 or 297.*

**Reply2: We have now added the name of the test together with the p-value, if not indicated. Full table of the statistical values is described in table S1.**
